# Spontaneous remission of retinal capillary macroaneurysm: case report

**DOI:** 10.3389/fmed.2025.1567832

**Published:** 2025-10-23

**Authors:** Shui Lu, Xiaomei Meng, Tiantian Chen, Zhengwei Zhang

**Affiliations:** ^1^Department of Ophthalmology, Jiangnan University Medical Center, Wuxi, China; ^2^Department of Ophthalmology, Wuxi No.2 People's Hospital, Affiliated Wuxi Clinical College of Nantong University, Wuxi, China

**Keywords:** retinal capillary macroaneurysms, perifoveal exudative vascular anomalous complex, PEVAC, telangiectatic capillaries, TelCaps

## Abstract

**Background:**

Retinal capillary macroaneurysms (RCM), a rare macular vascular disease, was reported here for the first time in a Chinese patient.

**Case presentation:**

A 68-year-old female patient presented with a complaint of blurred vision in her right eye for over a month. Upon specialized examination, her best-corrected visual acuity (BCVA) was 20/50 in the right eye. Fundus examination revealed yellow-white, hard exudates in the nasal and temporal sides of the fovea and a suspected hemorrhagic lesion was observed external to the inferior vascular arcade. Optical coherence tomography (OCT) scanning through the foveal center revealed a serous neurosensory detachment with intraretinal hard exudates. A preliminary diagnosis of chronic branch retinal vein occlusion (BRVO) in the right eye was made. To alleviate the macular serous detachment, the treatment plan was intravitreal injection of conbercept once a month. One month after the first injection, the patient reported worsening symptoms. BCVA in the right eye decreased to 20/100, and fundus examination revealed a ring-shaped yellow-white, fine, hard exudates surrounding the initial lesion. Two more intravitreal injections were given one month apart. Subsequently, intravitreal injections were discontinued due to inadequate therapeutic efficacy and considerations regarding treatment costs. Approximately 10 months after the last intravitreal injection, the patient reported improved vision. Optical coherence tomography angiography (OCTA) revealed a vertical oval tubular structure with a hyperreflective wall external to the inferior vascular arcade, with blood flow signals at the top of the lumen. Six more months later, the patient reported stable vision. OCTA still showed a vertical oval tubular structure, and the lumen diameter had increased. One more year later, the patient had a follow-up and the BCVA was 20/20 in both eyes. OCTA showed complete closure of the aneurysm without any abnormal blood flow. The right eye was conclusively diagnosed with RCM.

**Conclusion:**

This report presented the first documented Chinese case of isolated RCM. Although the lesion initially enlarged after three intravitreal injections of conbercept, it eventually resolved spontaneously. This outcome suggested that close observation may be appropriate for RCM patients with stable, good vision if such patients are unwilling or afraid to receive laser treatment.

## 1 Introduction

Retinal capillary macroaneurysm (RCM), first described by Spaide et al. in 2019, is an unusual type of large aneurysm that originate from retinal capillaries, characterized by isolated lesion and over time growing larger in size (1). They included five cases with aneurysms that reached a maximum mean size of 273.4 μm: four patients had no other discernable retinal vascular disease, and one patient had stable diabetic retinopathy (DR). In other words, the pathogenesis of RCMs is poorly understood and often occurs in isolation. Anti-vascular endothelial growth factor (anti-VEGF) treatment was associated with a partial response in one patient, while laser photocoagulation of the aneurysms led to the resolution of edema and the disappearance of the lesions in all patients. Here, we reported a Chinese patient who developed RCM that grew larger after receiving three intravitreal injections of conbercept (0.5 mg) due to subretinal fluid (SRF) in the macula. Notably, in this case, the RCM spontaneously resolved without any subsequent treatment over a 30-month follow-up period. This case study can be valuable in determining the appropriate treatment strategy for RCM based on the specific clinical characteristics of each case.

## 2 Case presentation

A 68-year-old female presented to the Department of Ophthalmology, Jiangnan University Medical Center on April 4, 2018, complaining of blurred vision in her right eye for over a month. Her medical history included hypertension for 10 years, which was well-controlled with oral medication. She reported no history of diabetes mellitus or any other chronic systemic diseases. She also denied engaging in harmful habits, such as smoking, and confirmed the absence of any family history of ocular diseases.

**Figure 1:**
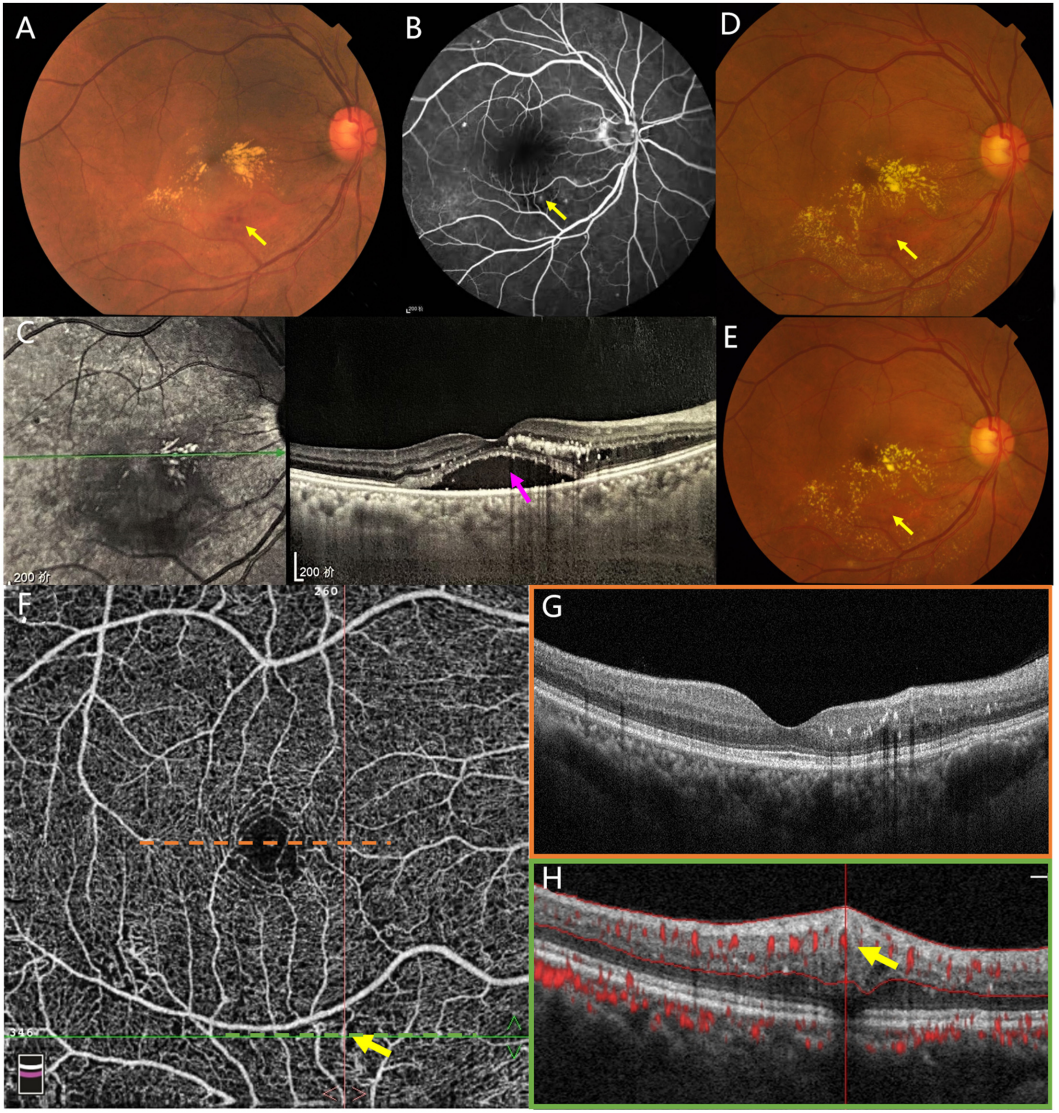
On initial examination, color fundus photography revealed yellowish-white hard deposits in the macular area. A suspected hemorrhagic lesion was observed inferior to the macula [**(A)**, yellow arrow]. Fundus fluorescein angiography (FFA) demonstrated a corresponding punctate hyperfluorescent lesion at the site [**(B)**, yellow arrow]. The remaining hyperfluorescent spots were confirmed by optical coherence tomography as small dome-shaped retinal pigment epithelial detachment (PED). Optical coherence tomography (OCT) through the fovea showed subretinal fluid (SRF, pink arrow) with dense intraretinal hard exudates **(C)**. One month after the first intravitreal injection of conbercept, color fundus photography showed progression of hard exudates, which formed a ring centered on the original lesion **(D)**. One month after the third intravitreal injection of conbercept, color fundus photography revealed partial resolution of the ring of hard exudates **(E)**. Yellow arrows indicate the location of the lesion throughout the images. Optical coherence tomography angiography (OCTA) revealed an anomalous, sausage-shaped dilated blood flow signal (yellow arrow) external to the inferior vascular arcade **(F)**. Optical coherence tomography (OCT) imaging through the fovea demonstrated complete resolution of subretinal fluid (SRF) with sparse intraretinal hard exudates [**(G)**, orange box]. A cross-sectional OCT scan showed a vertically elliptical, tubular structure within the middle retina, characterized by a hyperreflective wall, a medium-to-low reflective lumen, and blood flow at the apical portion of the lumen [**(H)**, green box, yellow arrow].

Initial examination: Best-corrected visual acuity (BCVA) was 20/50 in the right eye and 20/20 in the left eye. Both eyes had normal intraocular pressure, clear corneas, and a lens with mild cortical opacity. Fundus examination of the right eye revealed yellowish-white, granular hard exudates on the nasal and temporal sides of the fovea. A suspected hemorrhagic lesion was noted temporal to the inferior retinal vascular arcade of the fovea (Figure 1A). Fundus fluorescein angiography (FFA) demonstrated a punctate hyperfluorescent lesion corresponding to the suspected hemorrhage (Figure 1B). A cross-sectional optical coherence tomography (OCT) scan through the fovea showed serous retinal detachment with dense intraretinal hard exudates (Figure 1C). A provisional diagnosis of chronic branch retinal vein occlusion (BRVO) was considered, despite the patient reporting no history of abnormal vision in her right eye. However, retinal artery filling time was normal, and cervical Doppler ultrasonography showed no significant abnormalities. Upon multimodal imaging examination, no significant abnormalities were detected in the left eye.

Treatment and subsequent follow-ups: Intravitreal injections of conbercept (0.5 mg/0.05 ml) were administered monthly to reduce macular edema. One month after the first injection (May 5, 2018), the patient reported worsening symptoms, with her BCVA in the right eye declining to 20/100. Fundus examination revealed a ring-shaped, yellowish-white, fine granular hard exudates centered around the initial lesion (Figure 1D). Two more intravitreal injections were administered over the next two months. One month after the third injection (July 2, 2018), the patient reported slight improvement in blurred vision, with her BCVA in the right eye improving to 20/60. Fundus examination showed a large aneurysm with white rim and partial absorption of the ring-shaped hard exudates (Figure 1E). Subsequently, the patient declined further intravitreal injections and failed to adhere to the prescribed follow-up schedule due to the inadequate therapeutic efficacy and high cost of the treatment. The patient also declined laser treatment after a discussion of the potential risks, which included retinal hemorrhage and a possible decrease in visual acuity.

**Figure 2:**
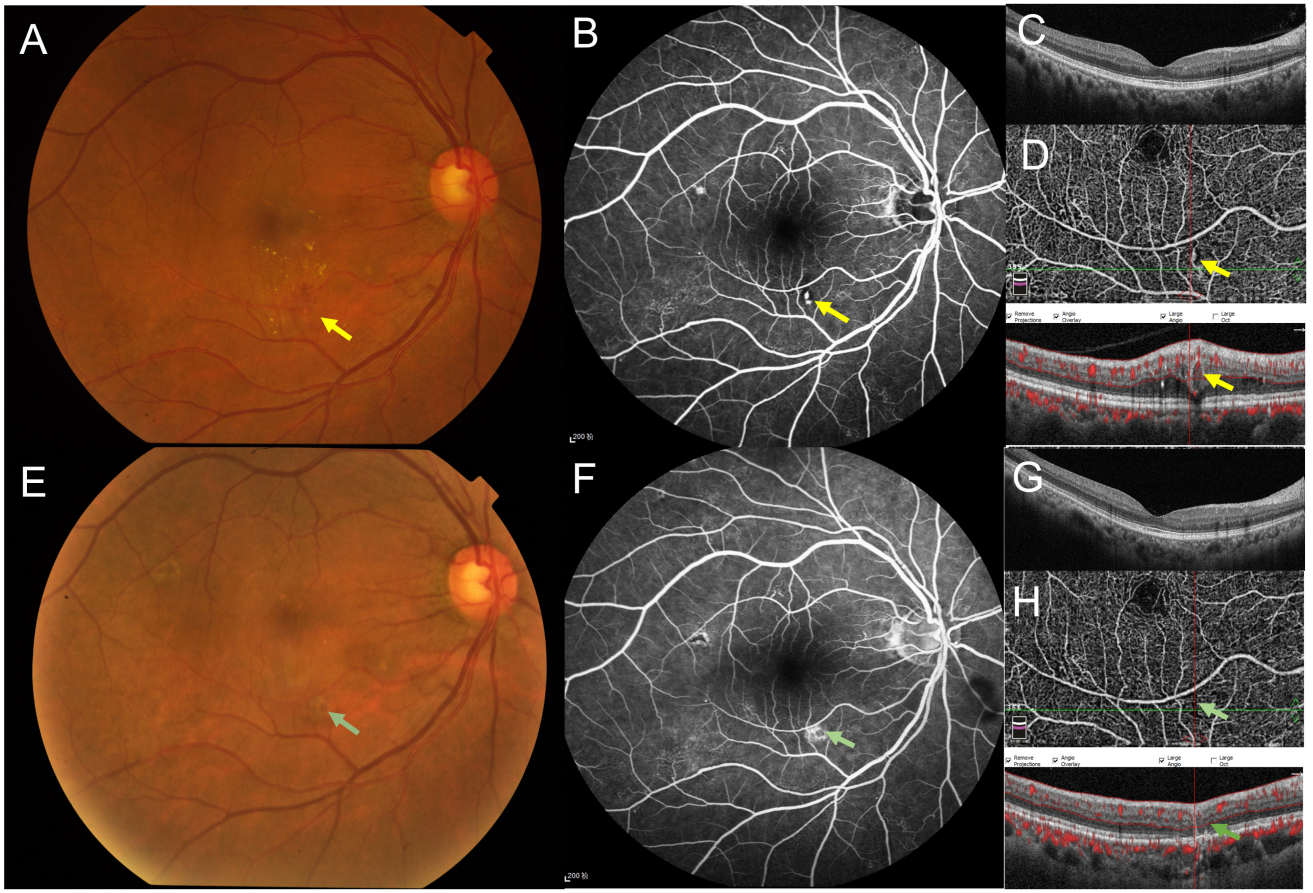
Follow-up at 1.5 years after initial presentation, color fundus photography showed almost complete resolution of the ring-shaped hard exudates **(A)**. Fundus fluorescein angiography (FFA) revealed an irregular, punctate hyperfluorescent lesion [**(B)**, yellow arrow]. Optical coherence tomography (OCT) imaging through the fovea demonstrated complete resolution of subretinal fluid (SRF) with few intraretinal hard exudates **(C)**. Optical coherence tomography angiography (OCTA) continued to show an anomalous, sausage-shaped dilated blood flow external to the inferior vascular arcade, with a significantly increased lumen diameter compared to Figure 1H on the cross-sectional OCT [**(D)**, yellow arrow]. Follow-up at nearly 3 years after initial presentation, color fundus photography showed complete resolution of the ring-shaped hard exudates **(E)**. FFA demonstrated fluorescein staining at the site of the original lesion [**(F)**, green arrow]. OCT imaging through the fovea revealed complete resolution of SRF without any intraretinal hard exudates **(G)**. OCTA showed complete disappearance of the anomalous, sausage-shaped dilated blood flow signal, and a corresponding cross-sectional OCT scan confirmed complete disappearance of the oval lesion [**(H)**, green arrow].

Long-term follow-up: Approximately 10 months after the last intravitreal injection (April 23, 2019), the patient reported improved vision with a BCVA of 20/50 in the right eye. Fundus examination showed further absorption of the hard exudates. Optical coherence tomography angiography (OCTA) demonstrated an anomalous, sausage-shaped dilated blood flow signal (Figure 1F) and the corresponding cross-sectional OCT scan through the lesion revealed a vertical, oval, tubular structure characterized by hyperreflective wall external to the inferior vascular arcade, with blood flow visible at the top of the lumen (Figure 1H). At this juncture, a cross-sectional OCT scan through the fovea demonstrated complete resolution of SRF (Figure 1G). Six more months later (October 22, 2019), the patient reported stable vision and no discomfort. BCVA had improved to 20/25 in the right eye and remained 20/20 in the left eye. Fundus examination revealed significant absorption of the ring-shaped hard exudates (Figure 2A). FFA demonstrated an irregular, punctate hyperfluorescent lesion external to the inferior vascular arcade without late leakage (Figure 2B). A cross-sectional OCT scan through the fovea showed no evidence of macular edema (Figure 2C). OCTA continued to display a vertical, oval, tubular structure external to the inferior vascular arcade, but with an enlarged lumen (Figure 2D). One more year later (November 27, 2020), the patient reported stable vision with a BCVA of 20/20 in both eyes. Fundus examination showed complete resolution of the ring-shaped hard exudates (Figure 2E). FFA revealed disappearance of the punctate hyperfluorescence, with late dye staining (Figure 2F). A cross-sectional OCT scan through the fovea continued to show no evidence of macular edema (Figure 2G). OCTA showed complete disappearance of the anomalous, sausage-shaped dilated blood flow, and a corresponding cross-sectional OCT scan confirmed complete disappearance of the oval lesion (Figure 2H).

Diagnostic assessment: The initial diagnosis of BRVO was challenged by several key findings. First, the patient exhibited no characteristic retinal hemorrhages typically associated with BRVO. Second, neither FFA nor OCTA revealed significant areas of non-perfusion, which are commonly linked to BRVO-related large retinal capillary aneurysms. Third, unlike the macular edema typically seen in BRVO, the patient's visual acuity did not improve following three intravitreal anti-VEGF injections, and the annular hard exudates became more pronounced. Notably, the center of these exudates often corresponds to the location of underlying retinal vascular abnormalities. In this case, OCTA provided definitive evidence by clearly visualizing sausage-shaped dilated capillaries and confirming the absence of surrounding non-perfusion. These findings collectively supported the conclusion that the abnormal retinal capillary aneurysm was idiopathic rather than secondary to BRVO, leading to a final diagnosis of RCM. The clinical timeline for the patient was summarized in Table 1.

**Table 1:** Timeline of the patient's care and follow-up.

**Time point**	**Key events and interventions**	**Clinical outcomes and examination data**
April 2018	Admission for blurred vision in her right eye for over a month; initial diagnosis: BRVO (OD) Three intravitreal injections of conbercept (0.5 mg) per month	BCVA: 20/50 (OD), 20/20 (OS); hard exudates and a suspected hemorrhagic lesion in the macula of right eye (Figure 1A); a punctate hyperfluorescent lesion on FFA (Figure 1B); SRF with dense intraretinal hard exudates on structural OCT (Figure 1C)
May 2018	One month after the first injection of conbercept (0.5 mg)	BCVA: 20/100 (OD), 20/20 (OS); a ring-shaped, yellowish-white, fine granular hard exudates centered around the initial lesion (Figure 1D)
July 2018	One month after the third injection of conbercept (0.5 mg)	BCVA: 20/60 (OD), 20/20 (OS); a large aneurysm with white rim and partial absorption of the ring-shaped hard exudates (Figure 1E)
April 2019	1-year follow-up without more intervetions	BCVA: 20/50 (OD), 20/20 (OS); an anomalous, sausage-shaped dilated blood flow signal on OCTA (Figure 1F); complete resolution of SRF on structural OCT (Figure 1G); cross-sectional OCT scan through the lesion revealed a vertical, oval, tubular structure characterized by hyperreflective wall (Figure 1H)
October 2019	1.5-year follow-up without more intervetions	BCVA: 20/25 (OD), 20/20 (OS); significant absorption of the ring-shaped hard exudates (Figure 2A); an irregular, punctate hyperfluorescent lesion on FFA (Figure 2B); no evidence of macular edema on OCT (Figure 2C); a vertical, oval, tubular structure on OCTA (Figure 2D)
November 2020	2.5-year follow-up without more interventions Final diagnosis: RCM (OD)	BCVA: 20/20 (OD), 20/20 (OS); complete resolution of the ring-shaped hard exudates (Figure 2E); disappearance of the punctate hyperfluorescence, with late dye staining on FFA (Figure 2F); no evidence of macular edema on OCT (Figure 2G); complete disappearance of the vertical, oval, tubular structure both on OCT and OCTA (Figure 2H)

BCVA, best-corrected visual acuity; OD, right eye, OS, left eye; BRVO, branch retinal vein occlusion; FFA, fundus fluorescein angiography; OCT, optical coherence tomography; OCTA, optical coherence tomography angiography; RCM, retinal capillary macroaneurysm.

## 3 Discussion

Microaneurysms arising from retinal capillaries are commonly observed in various retinal diseases, such as DR and retinal vein occlusion (2, 3). However, larger capillary aneurysms, often defined as those exceeding 100–150 μm in diameter, are also not uncommon. Due to the lack of a standardized nomenclature (4), these lesions have been variously termed: large capillary aneurysms (5), larger capillary aneurysms (6) macro-microaneurysms (7), capillary macroaneurysms (CMAs) (8–10), telangiectatic capillaries (TelCaps) (11–13), intraretinal macroaneurysms (IMAs) (14) and retinal capillary macroaneurysms (RCMs) (1). Among these terms, RCMs are specifically used to describe solitary, relatively large, tumor-like lesions arising from retinal capillaries, in the absence of other known retinal vascular diseases. Recently, RCMs have also been reported in cases involving diabetes mellitus, both with and without DR (15–17). While TelCaps were first described by Chew et al. in 1986 and were originally used to describe telangiectatic capillaries in DR (18), they have also been used in cases secondary to other retinal diseases, such as RVO (19–21) and radiation retinopathy (22). Despite the lack of consensus, we propose using RCMs as the term with the least ambiguity. For this reason, our case was diagnosed using this nomenclature.

In recent years, there has been increasing interest in a specific idiopathic retinal vascular disorder known as perifoveal exudative vascular anomalous complex (PEVAC) (23). The debate persists as to whether PEVAC is essentially a type of RCMs (24, 25). From an imaging perspective, PEVAC lesions exhibit characteristic features on OCT. These include a vertical, oval tubular structure with a hyperreflective wall and a heterogeneous hypo- to mid-reflective lumen. Blood flow can be detected at the apex of the lumen on OCTA (26). However, these imaging findings are not exclusive to PEVAC, as similar appearances can be observed in large retinal aneurysms (27).

Unlike TelCaps, which are often used to describe retinal capillaries dilation with underlying retinal vascular diseases like DR or RVO (11–13), RCMs and PEVAC are generally considered isolated, benign, and relatively large tumor-like growths of the retinal microvasculature. The primary distinction between the two lies in the location, as RCMs can occur anywhere in the macular region, not being confined to the perifoveal area [7]. Both terms share many clinical similarities, including a predilection for eyes without a clear underlying etiology, typically presenting as solitary, vascular tumor-like lesions. Additionally, both terms demonstrate a poor response to intravitreal anti-VEGF injections and may or may not be accompanied by hard exudates (1, 28). Besides, previous studies have shown that PEVAC may remain stable or even spontaneously resolve over time (29), whereas RCMs tend to increase in size (1). Here, we present the first reported case of spontaneous remission in an RCM. The clinical features of RCM, PEVAC, and TelCaps are listed in Table 2.

**Table 2:** Clinical features of RCM, PEVAC, and TelCaps.

**Clinical features**	**RCM**	**PEVAC**	**TelCaps**
Lesion characteristics	Occurs in the macular region but is not confined to the perifoveal area, with or without hard exudates or hemorrhage	Mainly confined to the perifoveal area, with or without hard exudates or hemorrhage	Occurs in the macular region but is not confined to the perifoveal area, with or without hard exudates or hemorrhage
FFA findings	Solitary, relatively large, tumor-like lesions arising from retinal capillaries	Solitary, relatively large, tumor-like lesions arising from retinal capillaries	Solitary or multifocal, relatively large, tumor-like lesions arising from retinal capillaries
OCT and OCTA findings	An aneurysm-like OCTA lesion originating from the retinal capillary bed, typically measuring ≥100 μm in diameter, and characterized by a hyperreflective wall with a hyporeflective lumen on OCT B-scan		
Underlying cause	Idiopathic or in the context of diabetes	Idiopathic or in the context of other retinal diseases, including diabetes, age-related macular degeneration, and pathologic myopia	Secondary to retinal vein occlusion or diabetic retinopathy
Treatment response	Poor response to intravitreal anti-VEGF injections; low-power short-duration thermal focal laser is effective for extrafoveal lesion	Poor response to intravitreal anti-VEGF injections; the laser is not suitable for lesion near the central fovea	Poor response to intravitreal anti-VEGF injections; low-power short-duration thermal focal laser is effective for extrafoveal lesion

RCM, retinal capillary macroaneurysm; PEVAC, perifoveal exudative vascular anomalous complex; TelCaps, telangiectatic capillaries; OCT, optical coherence tomography; OCTA, optical coherence tomography angiography.

In our case, although the patient had a history of hypertension, it was well-controlled with medication, and other systemic chronic diseases were ruled out. In contrast to conventional BRVO, which manifests with conspicuous retinal surface hemorrhages, this case demonstrated an absence of typical retinal surface bleeding throughout its course. Due to the uncertain initial diagnosis of RCM, and despite the atypical clinical features for a conventional BRVO, the patient was initially misdiagnosed and treated as a BRVO case. However, three intravitreal anti-VEGF injections failed to produce the desired therapeutic outcome. Because of the high cost of treatment, the patient subsequently declined further interventions, expressing a loss of confidence in the therapeutic approach. Fortunately, the patient remained under our care with aperiodic follow-up visits, and the final outcome was a surprisingly favorable spontaneous resolution. Retrospective analysis of this case ultimately elucidated the underlying etiology for the SRF, and hard exudates noted at the initial consultation.

Specifically, color fundus photography showed a progressive development of a circular hard exudate surrounding the lesion with white rim over time. A vascular lesion exhibiting a white rim may indicate a large retinal capillary aneurysm as recently reported in patients with diabetic retinopathy (6). FFA revealed an isolated, aneurysm-like lesion located away from the foveal avascular zone (FAZ), with no evidence of late leakage. OCTA demonstrated a sausage-like dilatation of capillary blood flow at the corresponding site. Structural OCT showed a gradual increase in the lesion diameter, which eventually resolved spontaneously by the last visit. Initially, three intravitreal anti-VEGF injections did not result in a rapid improvement in visual acuity and even caused a temporary decline. The ineffectiveness of anti-VEGF therapy further suggests that the pathogenesis of RCM may be similar to that of PEVAC and TelCaps, as they all involve intrinsic retinal microvascular abnormalities rather than neovascularization (30). However, the patient's vision improved gradually as SRF and hard exudates resolved over time, suggesting that the improvement in vision and resolution of the aneurysm were not directly related to the anti-VEGF treatment. Given the clinical findings and the lesion's distance from the perifoveal area, a diagnosis of RCM was made. This case represented the first reported instance of spontaneous resolution of RCM in a Chinese patient. Our findings further support the hypothesis that RCM and PEVAC may represent different manifestations of the same underlying disease.

Regardless of whether RCMs are idiopathic or secondary to clear evidence of underlying cause, they tend to present with similar clinical features and OCT/OCTA findings. While intravitreal anti-VEGF injections are generally ineffective or partially effective for these lesions, laser photocoagulation can usually yield favorable outcomes (1, 9, 31), or be combined with intravitreal anti-VEGF injections (17). In a previous case report from our group (26), a patient with PEVAC experienced a subretinal hemorrhage after two intravitreal anti-VEGF injections, leading to a significant decrease in visual acuity. However, after complete resolution of the hemorrhage and exudates, the patient's BCVA recovered to 20/20. Therefore, for patients with RCMs, laser photocoagulation should be considered the first-line treatment, as other capillary dilation anomalies including PEVAC and TelCaps (12, 22, 31–34). Even if post-operative hemorrhage occurs, visual acuity can still improve after the resolution of the hemorrhage and SRF. In the present case, the patient's refusal of laser treatment due to its potential risks provided a unique opportunity for long-term observation without intervention. During our nearly three-year follow-up, despite the lesion's size continued enlargement, the hard exudates and SRF were largely absorbed, and the lesion eventually resolved spontaneously by the last visit. This result suggested that close observation may be appropriate for RCMs patients with stable, good vision if such patients are unwilling or afraid to receive laser treatment. Of note, this case report is limited by its single-patient observation, which may not apply universally, necessitating individualized clinical judgment to optimize patient outcomes.

## 4 Conclusions

In conclusion, this report presented the first Chinese case of isolated RCM. Although the lesion initially enlarged, it eventually resolved spontaneously, leading to the absorption of SRF and restoration of normal vision. For large aneurysms arising from retinal capillaries without a clear underlying etiology, we propose using the term “retinal capillary macroaneurysms.” Compared with PEVAC, this term not only accurately reflects the nature of the disease but also encompasses large, tumor-like lesions of retinal capillaries occurring outside the foveal area.

## Data Availability

The raw data supporting the conclusions of this article will be made available by the authors, without undue reservation.
